# Removal of bent intramedullary nail: Two case reports

**DOI:** 10.1097/MD.0000000000019935

**Published:** 2020-05-15

**Authors:** You-Sung Suh, Won-Seok Lee, Joonghyun Ahn, Hyung-Suk Choi, Min Jung Baek, Sung-Woo Choi

**Affiliations:** a Department of Orthopaedic Surgery, Soonchunhyang University Hospital Seoul; b Department of Orthopaedic surgery, Kyung Hee University hospital at Gangdong; c Department of Obstetrics and Gynecology, Bundang CHA Hospital.

**Keywords:** femoral fracture, implant removal, intramedullary nailing, metal failure

## Abstract

**Introduction:**

The removal of bent intramedullary (IM) nail can become a challenge. Therefore, various methods have been reported for the extraction of nails after femoral refracture. We want to share our successful treatment.

**Patient concerns:**

Case 1. A 44-year-old man was admitted to our clinic after falling while playing soccer. He complained severe right thigh pain with a visible deformity of the femur. His medical history revealed a right femoral shaft fracture caused in a traffic accident which had been treated with intramedullary nailing. Case 2. A 27-year-old man, who had suffered a right femur fracture after a motorcycle accident and been treated with an IM nail, presented after falling down the stairs. He had severe right thigh pain without any open wound or neurologic deficit.

**Diagnosis:**

Case 1. Plain radiographs revealed a refracture of the right femoral shaft and a bent IM nail. The initial varus deformity of the nail was 60.1° in the coronal plane. Case 2. The valgus deformity of the nail was 16.1° with an apex-posterior angulation of 34.8° in the sagittal image of plain radiographs.

**Interventions:**

Case 1. Initial manual reduction was tried in emergency room. Then, under general anesthesia closed reduction of the fracture and bent IM nail was done. After closed reduction, the nail was straightened and extracted smoothly. Case 2. Closed manipulation was attempted initially. But no difference in the deformity was achieved. Therefore, via skin incision, the bent nail was progressively sectioned with high-speed cutting burr until the nail could be straightened.

**Outcomes:**

Case 1. The patient was mobilized with partial-weight bearing assisted with a crutch on postoperative day two. One year after surgery, the fracture union was complete and the patient was pain-free. Case 2. Six months after surgery, the fracture union was complete with sufficient callus formation around the fracture site.

**Conclusion:**

There is no gold standard method to remove a bent IM nail. However, since manual reduction to straighten the bent nail causes minimal soft tissue damage, it should be considered first. If it fails, other methods should be attempted, progressing from the minimally invasive technique to more invasive methods.

## 1 Introduction

Intramedullary (IM) nailing is the current gold standard to treat femoral shaft fractures because of its low incidence of complications and high fracture union rate.^[1,2,3,4]^ The removal of IM nails is usually known to be a low-risk procedure with few complications.^[5]^ However, nail removal can become rather challenging when the nail is broken or bent. Particularly the latter can make the removal more difficult because the deformed nail cannot pass through the intramedullary canal.^[6,7]^ Therefore, a variety of methods have been reported for the extraction of bent IM nails after femoral refracture.^[7,8,9,10,11,12,13,14,15,16,17,18]^

In 1991, Patterson and Ramser^[16]^ described insitu straightening of a bent nail and some authors^[7,8,13]^ used percutaneous straightening and removal with a compression plate or steel drill without opening the fracture site. However, due to the stiffness of the nail, cases may require exposure of the fracture site and special cutting instruments to weaken or partially resect the nail.

We present two cases with bent IM nails after femoral refracture treated at our institution. We want to share our experience with the removal of these bent IM nails. One bent IM nail was removed after a closed manipulation, and the other was removed using a partial resection technique.

## 2 Case presentations

This case report was approved by the Institutional Review Board of Soonchunhyang University Hospital and the patient gave written informed consent for publication of this case report and accompanying images.

### 2.1 Case 1

**Figure 1:**
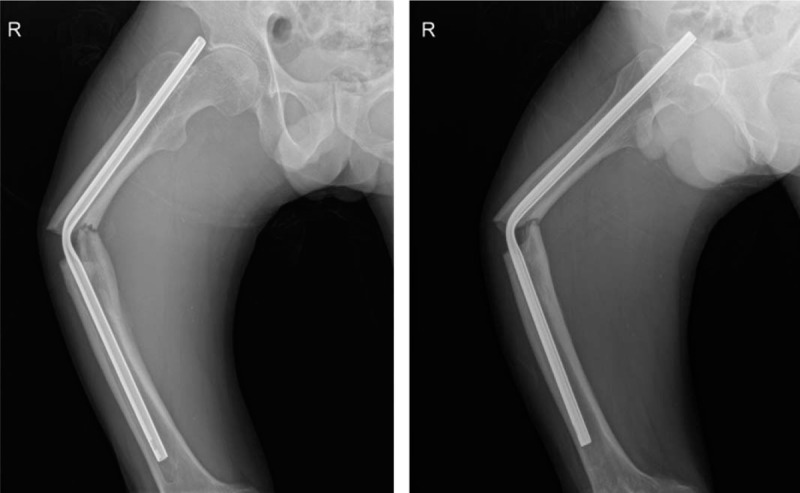
Radiographs showing a refracture of the right femoral shaft and a bent IM nail with 60.1° varus deformity.

**Figure 2:**
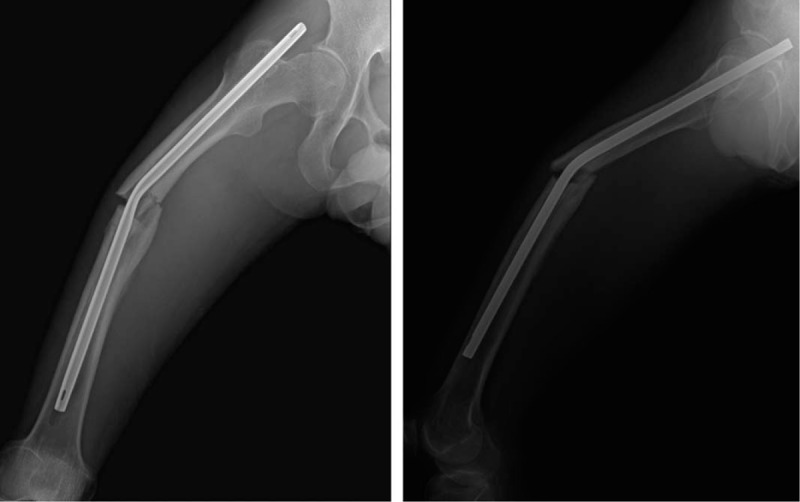
After initial reduction at emergency room, the varus deformity was decreased into 26.0°.

A 44-year-old man was admitted to our clinic after falling while playing soccer. The initial complaint was severe right thigh pain with a visible deformity of the femur. In our initial evaluation, there were no associated injuries. We could neither establish an open wound nor neurologic deficits. His medical history revealed a right femoral shaft fracture caused in a traffic accident approximately 22 years ago, which had been treated with intramedullary nailing at a hospital in China. Anteroposterior and lateral radiographs obtained in the emergency room revealed a refracture of the right femoral shaft and a bent IM nail (Fig. 1). The initial varus deformity of the nail was 60.1° in the coronal plane. We tried closed manual reduction of the fracture and bent nail with analgesics in the emergency room. After reduction, the varus deformity was decreased to 26.0° in the coronal plane (Fig. 2).

**Figure 3:**
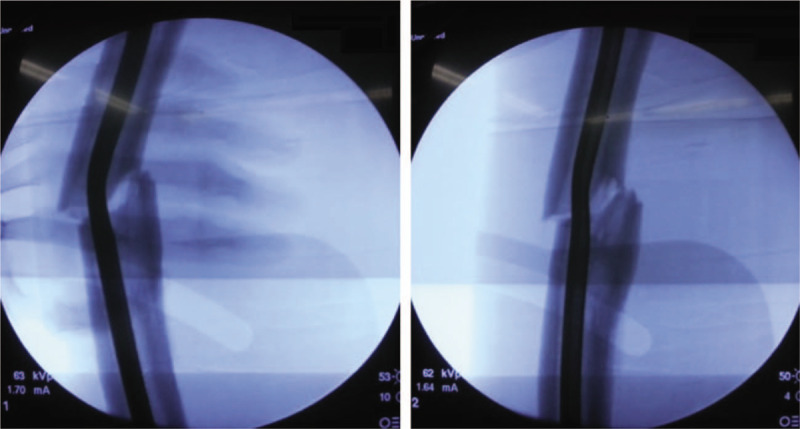
Fluoroscopic images showing a bent nail (A) and much corrected nail after closed manipulation (B).

**Figure 4:**
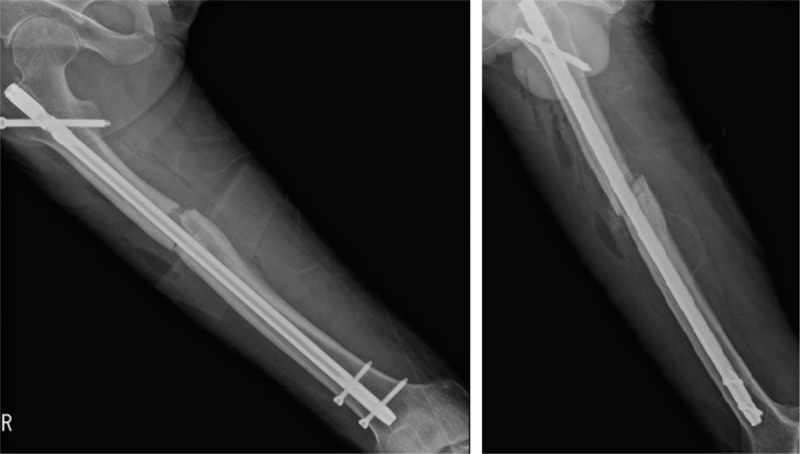
Radiographs after exchanging nailing using a new nail.

**Figure 5:**
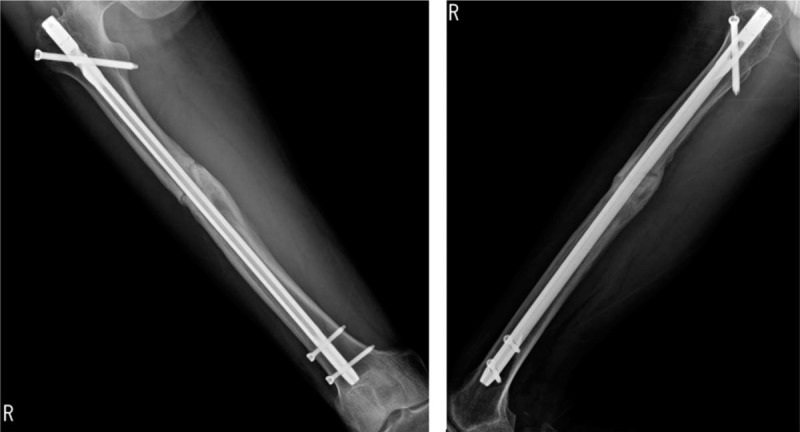
Radiographs showing the complete healing of the femoral shaft fracture.

Under general anesthesia, the patient was then placed in the left lateral decubitus position, and closed reduction of the fracture and bent IM nail was tried under C-arm image intensifier control. After the nail was straightened (Fig. 3), the entry site of the nail at the greater trochanter was exposed through a 5 cm skin incision. The nail was extracted smoothly by the standard method and in one piece. After nail extraction, the reaming of the intramedullary canal was performed to a diameter of 13 mm. A new IM nail (12 mm × 380 mm) was inserted through the previous insertion site and fixed with locking screws (Fig. 4). There were no postoperative complications and the patient was mobilized with partial-weight bearing assisted with a crutch on postoperative day two. One year after surgery, the fracture union was complete and the patient was pain-free (Fig. 5). The range of motion in the hip and knee were normal.

### 2.2 Case 2

**Figure 6:**
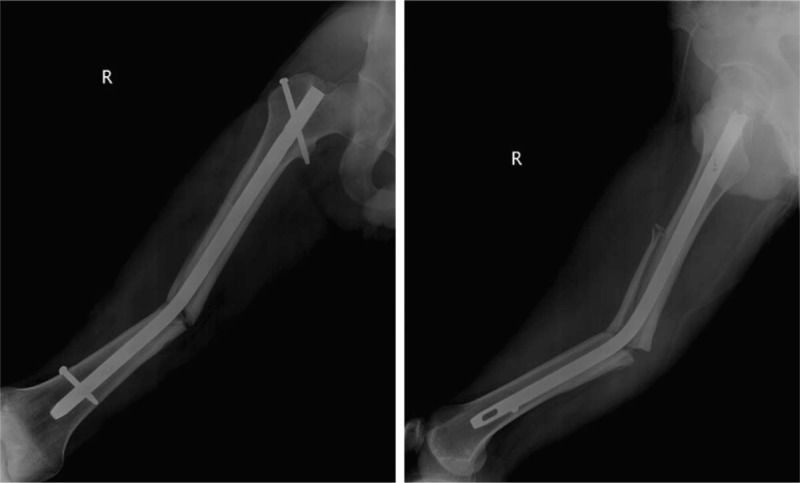
Radiographs showing bent IM nail with an apex-posterior angulation of 34.8° in the sagittal plane.

A 27-year-old man, who had suffered a right femur diaphyseal fracture after a motorcycle accident and been treated with an IM nail at our centre three years before, presented again after falling down the stairs. The incident had resulted in a refracture of the femoral shaft with posterolateral bending of the nail. The valgus deformity of the nail was 16.1° in the coronal plane with an apex-posterior angulation of 34.8° in the sagittal plane (Fig. 6). Removal of the nail and internal fixation were indicated.

**Figure 7:**
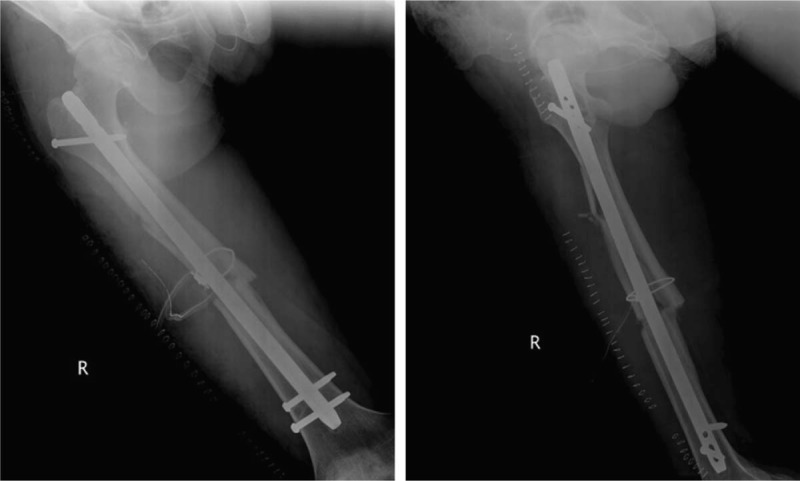
Radiographs after exchanging nailing using a new nail and a cerclage wire to fix the extended comminution of the fracture.

**Figure 8:**
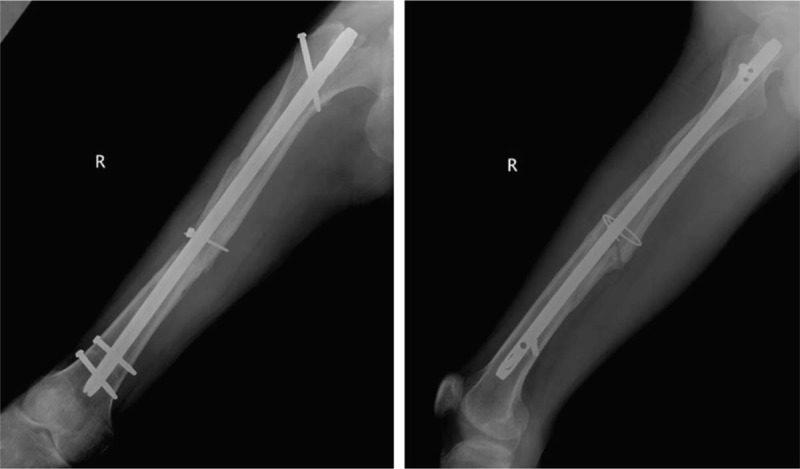
Radiographs showing the complete healing of the femoral shaft fracture.

The patient was placed in the left lateral decubitus position under general anesthesia. Initially, a closed manipulation of the bent nail was attempted under C-arm image intensifier control, but no difference in the valgus deformity was achieved. Intraoperatively, the skin was incised for a length of 15 cm above the bent part of the nail. Using a high-speed cutting burr, the apex part of the bent nail was progressively sectioned until the nail could be straightened. During this procedure, soft tissues were protected and permanently irrigated while any metal debris was carefully removed with the suction. Afterwards, the nail was extracted through the original incision at the hip. However, in the process of extracting the nail, the comminution of the fracture site was extended and had to be fixed with a cerclage wire. Then the femoral canal was reamed to 13 mm and a new nail (13 mm × 360 mm) was inserted (Fig. 7). No complications occurred postoperatively. Six months after surgery, the fracture union was complete with sufficient callus formation around the fracture site (Fig. 8).

## 3 Discussion

Bending of IM nails is most commonly linked to trauma, while breakage of the nail mostly occurs with nonunion, unstable, or pathologic fractures because of metal fatigue, and/or thin nails.^[10,19]^ The currently used titanium alloy IM nails have a lower elastic modulus than conventional stainless steel nails, but have better biomechanical stability after insertion. Also, because stainless steel shows high stiffness, stress shielding can occur during the fracture healing process.^[20]^ Therefore, bone loss can progress even after the fracture has healed, and, if the appropriate strength is not maintained, there is a high possibility that the secondary external force will cause fracture and/or deformation of the nail.^[21,22,23,24]^ There are cases where stainless steel nails are not routinely removed after healing of the fracture. Consequently, the fracture site may not be built to appropriate strength due to the ongoing bone loss from stress shielding, and this, in combination with fatigue of the metal, is then thought to result in shaft refracture with bending of the nail.

The extraction of a bent nail is more complicated than removing a broken nail because nail deformation usually results in blockage of the intramedullary canal.^[6,7]^ There is no generally accepted method for the removal of a bent IM nail, and various techniques have been proposed.^[7,8,9,10,11,12,13,14,15,16,17,18]^ They may be distinguished into two main categories, one is to reduce the fracture and the bent nail after exposure of the fracture site, and the other is not. Many different instruments are used to reduce and extract the nail. Table 1 provides a summary of the various studies describing different methods for nail removal.

**Table 1:**
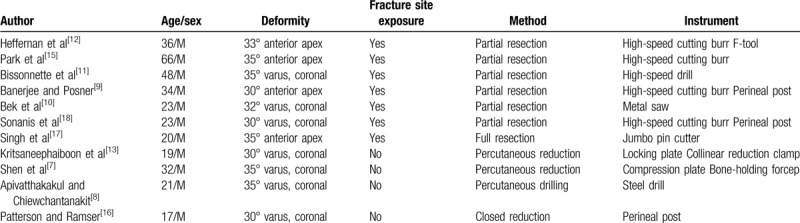
Summary of removal techniques of bent IM nail (previously described techniques).

Manual reduction may not result in adequate reduction if the nail is strong. In this case, using a compression plate together with a reduction clamp is a well-known method as it allows to apply maximum strength to correct the deformity. In addition, the temporary stabilization of the fracture site with the plate and clamps decreases the risk of iatrogenic fracture during nail removal, a complication we observed in our second case. In case this method does not achieve reduction, or where there is extreme deformity, percutaneous drilling or resection of parts of the nail with a cutting burr should be performed to weaken the nail. However, these procedures require special cutting equipment, and thermal injury, metal debris, and soft tissue damage occurring in the process may interfere with the consecutive fracture healing process.

In our first case, wound healing was completed within two weeks after insitu reduction. However, in the second case, where we had to resect the nail by cutting it into pieces, redness around the wound persisted for two weeks and surgical site pain for about a month. Metal debris can cause pain in the soft tissue around the implant, activate the patient's immune system, delay wound healing, and even induce necrosis.^[25,26]^ Therefore, if cutting or drilling of the nail is performed, it is critical to remove metal debris by appropriate irrigation and suctioning. Apivatthakakul et al. performed irrigation and suctioning through the drill sleeve during percutaneous drilling,^[8]^ whereas Banerjee et al. used surgical lubricant to protect the soft tissue from the accumulation of metal debris.^[9]^

In our opinion, the first case of this study, where the bent IM nail was straightened by manual reduction, probably represents the least soft tissue injury and fracture healing interference. Therefore, this method should principally be considered first. If it fails, we recommend to attempt reduction with a minimally invasive technique such as the plate and reduction clamp with percutaneous drilling before resorting to more invasive methods such as nail resection with a cutting burr or blade.

## 4 Conclusion

There is no gold standard method to remove a bent IM nail. However, since manual reduction to straighten the bent nail causes minimal soft tissue damage, it should be considered first. Only if it fails, other methods should be attempted, progressing from the minimally invasive reduction technique with a plate and reduction clamp and percutaneous drilling to more invasive methods like nail resection with a cutting burr or blade.

## Author contributions

**Conceptualization:** Sung-Woo Choi, Joonghyun Ahn.

**Data curation:** Hyung-Suk Choi.

**Funding acquisition:** Sung-Woo Choi.

**Project administration:** You-Sung Suh.

**Supervision:** You-Sung Suh.

**Visualization:** Joonghyun Ahn, Won-Seok Lee.

**Writing – original draft:** Won-Seok Lee, Min Jung Baek.

**Writing – review & editing:** Hyung-Suk Choi.
